# Goal Attainment Scaling in Outpatient Physical Therapy for Chronic Low Back Pain: Protocol for a Mixed Methods Study

**DOI:** 10.2196/32457

**Published:** 2022-03-07

**Authors:** Douglas Haladay, Rebecca Edgeworth Ditwiler, Aimee B Klein, Rebecca Miro, Matthew Lazinski, Laura Lee Swisher, Jason Beckstead, Jay Wolfson, Dustin Hardwick

**Affiliations:** 1 School of Physical Therapy & Rehabilitation Sciences Morsani College of Medicine University of South Florida Tampa, FL United States; 2 College of Public Health University of South Florida Tampa, FL United States

**Keywords:** goal attainment scaling, goal setting, low back pain, chronic pain, physical therapy, patient engagement, adherence, rehabilitation, physical therapist

## Abstract

**Background:**

Patient engagement in decisions regarding their health care may lead to improved outcomes and improved adherence to treatment plans. While there are several options for involving patients in their health care, goal setting is a readily accessible method for physical therapists to increase the involvement of patients in health care decisions. Physical therapy goals are often generated by health care providers based on subjective information or standardized, fixed-item, patient-reported outcome measures. However, these outcome measures may not fully reveal the activity and participation limitations of individual patients. Goal attainment scaling (GAS) is a patient-centered approach that allows patients to set meaningful goals. While GAS has been shown to be reliable, valid, and sensitive to change in various populations, there is limited evidence in the United States on utilizing GAS in physical therapy for patients with chronic low back pain (LBP).

**Objective:**

The purpose of this paper is to describe the protocol for a study to (1) develop a way to apply GAS procedures for physical therapists treating patients with chronic LBP in the United States and (2) test the feasibility of applying GAS procedures for chronic LBP in an outpatient physical therapy setting.

**Methods:**

This study used a mixed methods design with 2 phases: qualitative and quantitative. The qualitative phase of the study employed focus groups of patients with chronic LBP to identify an inventory of goals that were important and measurable. A series of prompts was developed from this inventory to assist physical therapists in collaboratively establishing goals with patients in a clinical setting. The quantitative phase of the study pilot-tested the inventory developed in the qualitative phase in patients with chronic LBP to determine feasibility, reliability, validity, and responsiveness. We also plan to compare how well GAS reveals change over time relative to traditional, fixed-item, patient-reported measures.

**Results:**

Phase 1 data collection was completed in June 2020, while data collection for phase 2 was performed between March 2021 and December 2021. We anticipate that this study will demonstrate that GAS can be implemented successfully by outpatient physical therapists, and that it will demonstrate clinically important changes in patients with chronic LBP.

**Conclusions:**

GAS represents an opportunity for patient-centered care in the physical therapy management of chronic LBP. While GAS is not new, it has never been studied in real-world physical therapy for chronic LBP in a clinical setting. Due to unique time and productivity constraints, for GAS to be successfully implemented in this environment, we must demonstrate that clinicians can be trained efficiently and reliably, that GAS can be implemented in a clinical setting in under 15 minutes, and that GAS is able to detect clinically meaningful changes in patient outcomes.

**International Registered Report Identifier (IRRID):**

DERR1-10.2196/32457

## Introduction

The patient experience and patient-centered care are the core of the Institute for Healthcare Improvement’s “Triple Aim” (eg, population health, experience of care, and per capita cost) for optimizing the performance of health systems in the United States [1]. Patient engagement in decisions regarding their health care may lead to improved outcomes and improved adherence to treatment plans [2,3]. The interaction between the patient and provider is an essential element of patient-centered care [4]. The qualities of these interactions may best be judged by patients themselves. Patients value providers who listen to them, share information via dialogue, and consider their individual preferences in management of their health conditions [5]. In physical therapy, patients are satisfied if their physical therapist communicates effectively and spends adequate time explaining treatment options throughout the course of care [6]. Furthermore, outcomes and perceived quality of care may improve when patients are actively engaged in their own care [7].

While there are several options for involving patients in their health care, goal setting is a readily accessible method for physical therapists to increase the involvement of patients in health care decisions. Goal setting is an important part of physical therapy in episodes of care and in direct interventions, but the practice and implementation of goal setting is varied across the profession [8]. When physical therapists set goals, it is an opportunity to involve patients and to design interventions that consider individual patient needs [7].

Physical therapy goals are often provider generated and based on subjective information or standardized, fixed-item, patient-reported outcome measures [9]. However, these outcome measures may not fully reveal the activity and participation limitations of individual patients. As individuals have varied needs, their goals may not be identified by standardized measures, and therefore a patient’s particular goals may not be reflected in provider-directed goals. We have investigated patients’ views on whether pain, disability, and recovery measures are meaningful and have found that people with chronic low back pain (LBP) feel that standard measures used to classify patient goals do not reveal what is meaningful to them. Participants in focus groups often state that the standard outcome measures do not capture the fluctuating nature of symptoms or assess improvements in more active pursuits and often do not reveal the complex nature of social roles. While standardized outcome measures are useful for comparing populations, they may be of limited value when assessing individual patient-centered goals [9].

There are several patient-centered approaches used to involve patients in setting meaningful, individualized goals, including the Canadian Occupational Performance Measure, goal attainment scaling (GAS), and self-identified goals assessment [10-12]. GAS has been identified as one of the most time-efficient and reliable ways to involve patients in goal generation during clinical care in real-world settings [11]. GAS procedures are highly variable, with little consensus on the time needed to complete them in clinical practice. The reported time to complete the GAS process ranges from 5 minutes to 60 minutes [13-15]. This range in time to complete may be due to variations in GAS methods, such as the extent of patient involvement, family involvement, and whether GAS was completed by a team or an individual provider. In addition, setting specific goals may be more time-consuming in certain patient populations.

During GAS, patients are engaged in a dialogue to set specific, measurable, achievable, realistic, relevant, and time-based (SMART) goals [16]. Physical therapists often write goals using the SMART format and are well versed in writing SMART patient goals. The GAS procedure involves a discussion between the patient and provider about patient-directed goals and expected outcomes of treatment. This provides an opportunity for physical therapists to capture fluctuating pain levels, specific activities, and complex social responsibilities that are important to patients. The GAS process is readily accessible, free, and follows defined stages (ie, identifying goals, weighting goals, identifying expected outcomes, establishing a baseline score, and judging actual outcome versus expected outcome at follow-up) [11]. In contrast to standardized, fixed-item, patient-reported outcome measures, GAS generates a T score, which provides a numerical outcome of achievement that can be used for goals across the domains of the International Classification of Functioning, Disability, and Health (ICF) with varying difficulty, importance, and expected achievement [17]. The individualized outcome generated from GAS may prove helpful in enhancing traditional methods of collecting outcome measures and setting goals.

GAS is helpful when comparing heterogenous patient populations, who may have complex presentations and backgrounds [17]. Therefore, most of the literature related to applying GAS describes findings in a rehabilitation setting with pediatric patients or patients with neurological deficits [17,18]. Recently, GAS has been applied in patients with chronic LBP [2,9,19-22], as patients with chronic LBP have varying clinical presentations, severity levels, and treatment options [23]. Considering that LBP is the most common reason patients seek physical therapy in an outpatient setting [24] and that it is one of the most common causes of disability in the United States [25-27], patients with chronic LBP may be an ideal population for assessing the feasibility of GAS in a physical therapy outpatient setting. While there is evidence for the reliability, validity, and feasibility of GAS procedures [17], there is limited evidence in the United States about physical therapist use of GAS in the management of patients with chronic LBP. Furthermore, a recent systematic review found significant variability in GAS procedures used for patients with LBP and recommended development of a standardized approach and training for clinicians applying GAS [28]. GAS is a promising method of focusing on patient-centered outcomes and goals, but it is not clear how this method may complement standard, pre-existing outcome measures used by physical therapists and how feasible it is in an outpatient setting for patients with chronic LBP. GAS is novel because it provides a standardized means to set patient-provided goals that are quantifiable and can be used to track progress of the patient and compare outcomes across patients. Therefore, the purpose of this paper is to describe the protocol for a study to (1) develop a new application of GAS procedures to be used by physical therapists treating patients with chronic LBP in the United States and (2) test the feasibility of applying GAS procedures in the treatment of patients with chronic LBP in an outpatient physical therapy setting.

## Methods

### Ethics Approval

The University of South Florida (USF) Institutional Review Board approved this study on April 10, 2019 (Pro00035236), and the approval has been maintained in good standing.

### Study Design

**Figure 1:**
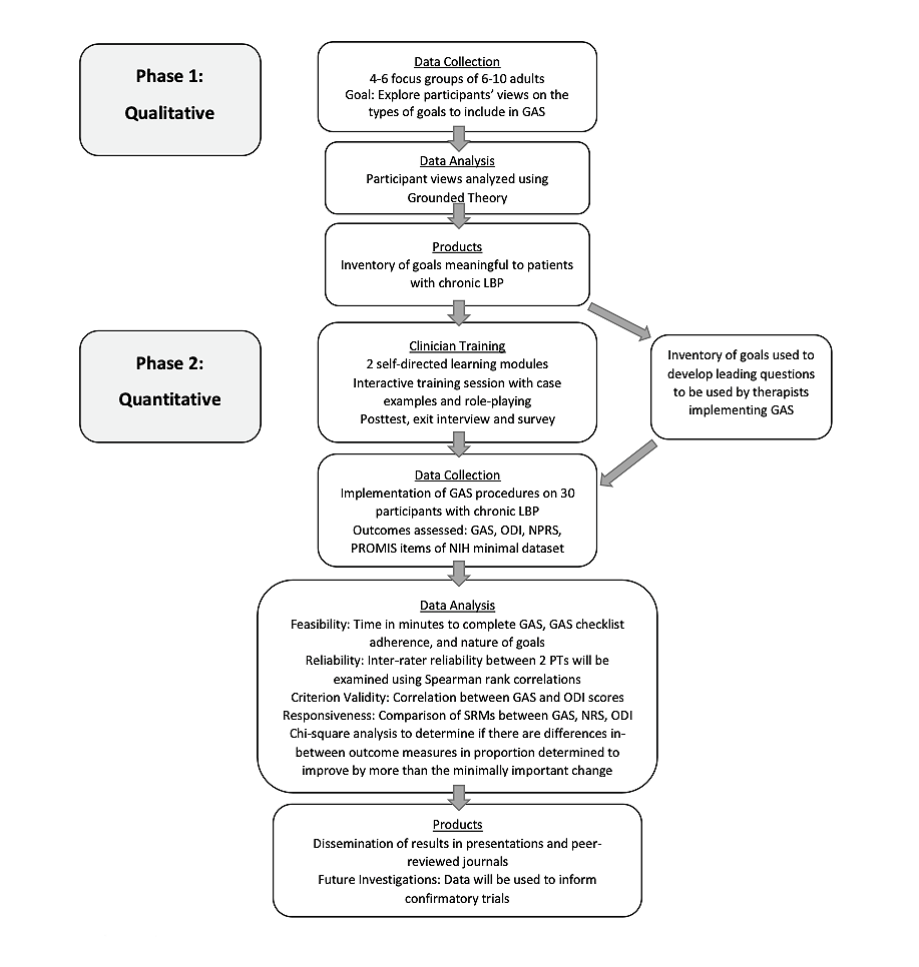
Study overview. GAS: goal attainment scaling; LBP: low back pain; NIH: National Institutes of Health; NPRS: numerical pain rating scale; ODI: Oswestry disability index; PROMIS: Patient-Reported Outcomes Measurement Information System; PT: physical therapist; SRM: standardized response mean.

This study used a mixed methods design with 2 phases: qualitative and quantitative. Figure 1 shows a study overview. The qualitative phase of the study employed focus groups of patients with chronic LBP to identify an inventory of goals that are important and measurable. This inventory was used to develop a series of prompts that will allow physical therapists to assist patients in establishing goals in a clinical setting [22]. The quantitative phase of the study pilot-tested the inventory developed in the qualitative phase in patients with chronic LBP to determine feasibility, reliability, validity, and responsiveness. We will also compare how well GAS identifies changes over time compared to traditional, fixed-item, patient-reported measures.

### Phase 1

#### Participants

We assembled 4 to 6 focus groups comprising 6 to 10 adults with chronic LBP from the local community using research alerts sent via the university email listservs. We expected that approximately 30 adults would be needed for this study, as a minimum of 4 focus group discussions are needed to reach code saturation [29-31]. Participants were included if they were adults (aged 21-64 years) with a history of nonspecific chronic LBP lasting >12 weeks with or without radicular symptoms. Participants were excluded if they had a structural spinal deformity, spinal fracture, osteoporosis, or systemic disease; had undergone previous spinal surgery; were pregnant or had given birth within the last 6 months; had pending litigation related to worker's compensation; or were undergoing treatment covered under worker's compensation. Participants were recruited from the Tampa Bay area.

#### Procedures

Focus groups were conducted face-to-face via internet-based meetings to explore participants’ views about the types of goals that should be included in GAS for patients with chronic LBP. There were 6 meetings with 6 to 10 participants that lasted approximately 2 hours [29]. An experienced facilitator led discussions. Field notes were taken during the interviews and audio recordings of each focus group were transcribed verbatim for further analysis.

#### Data Analysis

Participant views were examined with grounded theory principles [32,33]. A grounded theory approach was chosen because the intent of the qualitative portion of the study was to understand what was important about patients’ self-identified goals and why. This understanding was used to develop a standardized language and inventory of goals to facilitate the clinical implementation of GAS. We initially became familiar with the data by verifying the transcripts against the audio files and the field notes from each focus group to ensure accuracy and validation of speech allocation to individual participants. Coding began after the transcripts were read and became familiar to the researchers. Data were collected and coded until no new information was found (ie, saturation of the data was achieved). Qualitative data management software (MAXQDA, VERBI Software) was used to facilitate this process.

Once collected and transcribed, the data were independently coded, compared, and organized by 2 or more researchers using a constant comparison method. This qualitative procedure allows meaningful statements from the transcripts to be conceptualized in new ways [34]. The data coding process included open coding to determine code categories. Open coding was guided by sensitizing concepts found in the literature, including the ICF domains. Coding proceeded to the axial and selective phases to identify patterns in the data and identify the central themes that emerged. Several strategies were used to enhance rigor, including analytical triangulation using multiple coders, setting the goal for intercoder reliability to κ=0.80 (Cohen κ) using a coder-by-coder agreement matrix, peer debriefing group meetings to minimize researcher bias, and member checking to verify findings after focus group participation.

### Phase 2

#### Participants

We recruited approximately 30 patients with chronic LBP who sought physical therapy from the USF Physical Therapy Center at the USF Morsani Center for Advanced Healthcare. A sample of 30 participants is considered appropriate for pilot studies of feasibility, and sufficient to estimate effect sizes for confirmatory trials [35,36]. In order to estimate effect size, we used the findings from this study and retrospective change scores from patients with chronic LBP who did not participate in GAS (from our own clinic and from published data) as a comparison group. The USF Physical Therapy Center is the faculty practice of the USF School of Physical Therapy and Rehabilitation Science. This center services the Tampa Bay area and admits approximately 30 patients with chronic LBP each month. The demographics of Tampa Bay closely match national demographics and are very diverse socioeconomically, racially, and ethnically, making Tampa a strategically desirable location for clinical trials.

Participants who met the following criteria were sequentially recruited: aged 21-64, LBP located between the lower rib cage and gluteal fold [37], pain lasting >12 weeks [37-39], pain that was not attributable to a specific pathology [40], pain on at least 50% of days in the past 6 months [37], and average pain intensity >2 out of 10 on the Numerical Pain Rating Scale. Participants with a spinal deformity, surgery or fracture, rheumatoid arthritis, extremity pain, physical therapy treatment within the past 6 months, or automobile- or work-related injury were excluded.

#### Procedures

Physical therapists from the USF Physical Therapy Center were trained in GAS procedures based upon the principles of Williams and Stieg [2]; these procedures have been described for use in rehabilitation [41]. Therapist training consisted of 2 self-directed learning modules that covered background information, goal setting and negotiation, the benefits of GAS in diverse patient populations, the use of GAS in patients with chronic LBP, and the implementation of the stages of GAS. Case examples, including videos, were interwoven throughout the modules and an assessment was completed upon conclusion of training. The final step in the training was an interactive session with a study investigator that included role-playing the GAS procedures and providing feedback on performance. Once the therapists were trained (but before they saw patients as part of this study), they completed a posttest and a short survey and were briefly interviewed to examine their views regarding the feasibility of the GAS process. The training design will be streamlined for future studies to facilitate deployment to clinicians. Therapist interviews were transcribed for thematic analysis.

Once the therapists completed training and met all assessment criteria, therapist and patient encounters using GAS commenced. To recruit patients from the USF Morsani Physical Therapy Center, a clinician (included as a research staff member in this study) reviewed electronic medical records of incoming patients. Patients with chronic LBP were contacted via email or traditional mail (if a patient did not have or did not provide an email address) ahead of their visit with information regarding the study. This allowed the potential participant sufficient time prior to their first visit to consider whether they wished to take part in the study. Interested patients were instructed to contact members of the study team. Potential participants were screened and provided consent before participation. During the clinician encounter, which was part of their normal therapy visit, the patient and therapist jointly completed the first part of the GAS using the GAS-Back form. In addition, the participants self-reported the following measures: the Oswestry Disability Index, the Numerical Pain Rating Scale, and the National Institutes of Health Minimal Dataset. These patient-reported outcomes are commonly recommended for use in clinical and research settings and measure a patient’s perceptions of impairments in body structure and function, activity limitations, and participation restrictions (see Multimedia Appendix 1 for details) [37,42-44]. At the completion of the first visit, the patient completed a form for patient satisfaction with the goal-setting process and the clinician completed a form for patient level of engagement in goal setting. Audio recordings of patient visits were made. This was necessary to determine the reliability and feasibility of GAS in a clinical setting. Additionally, recording the encounter instead of having a research team member observing in the patient room allowed for a more natural interaction between clinician and patient. Following the initial visit, therapy sessions proceeded as determined in each participant’s physical therapy plan of care.

At the final physical therapy session (ie, at discharge), the Oswestry Disability Index and Numerical Pain Rating Scale were completed along with an abbreviated version of the National Institutes of Health Minimal Dataset, containing only the Patient-Reported Outcomes Measurement Information System items [37]. Additionally, the second part of the GAS-Back form, which regards goal achievement, was finalized, global perceived effect and patient satisfaction were measured, and the “collaboRATE” shared decision-making questionnaire was completed. We estimated these could be completed within 20 minutes. If a patient did not return for the last visit, the therapist called to follow up and the final questionnaire was completed over the phone or by using an online platform (Qualtrics).

#### Data Analysis

The success of GAS will be assessed based on feasibility, reliability, and validity. To consider the implementation of GAS in routine clinical practice, it must be administered in a timely (<15 minutes average examination time) and consistent (checklist adherence >80%) manner. The GAS process, feasibility, and fidelity will be evaluated by measuring the time (in minutes) to perform the GAS process, checklist adherence (as a percentage), and the nature of the goals identified (using the ICF domains) [45,46]. Interrater reliability of GAS scores will be assessed by examining the association between 2 independent physical therapist examiners using the Spearman rank correlation [33]. Criterion validity of GAS scores will be assessed by examining the association between GAS and Oswestry Disability Index scores [33,41]. The standardized response means (SRMs) for the Numerical Pain Rating Scale, Oswestry Disability Index, and GAS will be determined and compared to evaluate responsiveness [20]. A larger SRM indicates increased responsiveness of the measure to change. The SRM will be calculated as the ratio of change from pre- to posttest divided by the standard deviation of the change score [33]. A chi-square analysis will determine if different outcome measures show different proportions of patients determined to improve on that test by more than the minimally important change [33]. While a generally accepted and standardized definition of success for management of chronic LBP has not been established [42], our operational definition of success is as follows: if a patient shows changes that exceed the minimally important change or cutoff point for each individual measure, that patient’s outcome will be defined as successful. Minimally important changes for this analysis will be set at 2 points for the Numerical Pain Rating Scale [44], 10 points for the Oswestry Disability Index [47], and 2 points for the global perceived effect rating [48]. Cutoff points will be set at ≥50 points for GAS [20]. A GAS score of 50 indicates that the expected outcome was achieved, while a score greater than 50 indicates performance exceeding the expected outcome [20]. As analyses using change scores have weaknesses, we will also apply alternative approaches, such as analysis of covariance and residual change score [49]. The measures proposed in this study, including GAS, have acceptable reliability, validity, and responsiveness (Multimedia Appendix 1).

## Results

### Anticipated Results

Overall, we anticipate that this study will demonstrate that GAS can be implemented in a consistent and timely manner by outpatient physical therapists, and that patients with chronic LBP will demonstrate clinically important changes that are also important to them. In phase 1 we anticipate that the inventory of goals will accurately represent the domains most important to patients with chronic LBP. This inventory of goals should allow for a series of prompts that can be used by physical therapists to expedite the GAS process in an outpatient setting. In phase 2 we anticipate finding that GAS will be feasible to implement in an outpatient setting. To demonstrate this feasibility, we anticipate that we will find that physical therapist training results in a reliable and timely use of GAS. Furthermore, we anticipate that GAS will demonstrate validity and responsiveness to change when compared to outcome measures commonly used in chronic LBP.

### Study Timeline

#### Phase 1

Data collection was completed with the last focus group being held in June 2020. Preliminary data analysis was completed, and the information gleaned from this analysis was used to develop a series of prompts that were used to support the GAS process in phase 2.

#### Phase 2

Therapist training was completed in March 2021. Subject recruitment commenced following therapist training, and data collection began in March 2021. Data collection was completed in December 2021, and we expect data analysis to be completed by March 2022. This study is expected to conclude in late 2022.

## Discussion

### Principal Aims

This study aims to develop and test the feasibility of a novel application of GAS by physical therapists treating chronic LBP. It represents an important innovation because it facilitates patient-provider interaction and produces goals that encompass the activities and participation that are important to the patient. While goal setting is already part of the routine practice of physical therapists, the process is highly variable, with goals that are traditionally provider generated [8,50]. Knowledge of patient-initiated and patient-centered goals will enable health care providers to offer interventions that are more individualized and focused toward specific goals, leading to improved outcomes [18]. Furthermore, traditional, standardized, fixed-item patient-reported measures used for patients with chronic LBP (ie, the Numerical Pain Rating Scale and Oswestry Disability Index) may fail to measure constructs that are important to all patients, and therefore GAS may be better able to detect clinical changes that are meaningful to the patient. It is important to note that we are not recommending that we abandon current traditional, fixed-item, patient-reported outcome measures (eg, the Oswestry Disability Index and Numerical Pain Rating Scale). Rather, we believe that GAS can provide complementary information that augments these more established measures.

### Conclusion

GAS represents an opportunity for patient-centered care in the physical therapy management of chronic LBP. While GAS is not new, it has never been studied in real-world physical therapy for chronic LBP in a clinical setting, a type of practice that has unique time and productivity constraints. For GAS to be successfully implemented in this environment, we must demonstrate that clinicians can be trained efficiently and reliably, that GAS can be implemented in a clinical setting in under 15 minutes, and that GAS is able to detect clinically meaningful changes in patient outcomes.

## Appendix

Multimedia Appendix 1 Reliability and validity.

## Abbreviations

GAS: goal attainment scaling
ICF: International Classification of Functioning, Disability, and Health
LBP: low back pain
SMART: specific, measurable, achievable, realistic, relevant, and time-based
SRM: standardized response mean
USF: University of South Florida
