# Supplementary material for: Goal Attainment Scaling in Outpatient Physical Therapy for Chronic Low Back Pain: Protocol for a Mixed Methods Study
Source: JMIR Res Protoc. 2022 Mar 7;11(3):e32457. doi: 10.2196/32457 (PMC8938834; doi:10.2196/32457)
Supplement: Multimedia Appendix 1 [file resprot_v11i3e32457_app1.docx]

**Multimedia Appendix 1. Reliability and Validity:**

The measures proposed in this study, including Goal Attainment Scaling, have acceptable reliability, validity, and responsiveness.

| **Outcome Measurement Properties** | | | | |
| --- | --- | --- | --- | --- |
| **Measure** | **Domain** | **Reliability** | **Validity** | **Responsiveness** |
| Oswestry Disability Index^1, 2^ | ICF* Body Structure/Function, Activity, and Participation | Test-retest:  r = 0.83-0.99^1^  Internal Consistency: Chronbach’s α=0.71-0.87^1^ | Face and Content Validity^1^  Criterion-Related Validity: moderate correlation with pain (r = 0.62)^1^ | Minimally Import Change: 4.0-15 points^3^ |
| Numerical Rating Scale^3, 4^ | ICF* Body Structure/Function | Test-Retest:  r = 0.71-0.88^5^ | Predictive and Concurrent Validity^5^ | Minimally Import Change 1.0-4.5 points^3^ |
| Minimal Dataset^6^ | ICF* Body Structure/Function, Activity, and Participation | Unknown | Criterion-Related  Validity: moderate to strong correlation with Roland-Morris Disability Questionnaire (RMDQ)  (R=0.661) and Oswestry Disability Index (R=0.806)^6^ | Standardized Response Means (Change/SD of Change) indicate that impact stratification more responsive than RMDQ  (0.75 and 0.41, respectively)^6^ |
| Patient Satisfaction^7, 8^ | Satisfaction | Unknown | Criterion Validity  r = 0.72^9^ | Unknown |
| Global Perceived Effect (GPE)^10, 11^ | Generic | Test-retest:  ICC 0.90, 11 point scale^12^ | Face, Construct, and Concurrent Validity, 7 point and 15 point scales^12^ | Minimally Clinically Important Change 2 points for 11 point^12^ |
| Goal Attainment Scaling (GAS)^13, 14^ | Patient-Specific:  may span multiple domains | Inter-rater Reliability^15^ | Construct and Congruent Validity^15^ | More sensitive measure when compared to other outcomes^15, 16^ |

*International Classification of Functioning, Disability, and Health

References:

1. Fairbank JC, Pynsent PB. The Oswestry Disability Index. *Spine*. Nov 15 2000;25(22):2940-52; discussion 2952.

2. Fritz JM, Irrgang JJ. A comparison of a modified Oswestry Low Back Pain Disability Questionnaire and the Quebec Back Pain Disability Scale. *Physical therapy*. Feb 2001;81(2):776-88.

3. Ostelo RW, Deyo RA, Stratford P, et al. Interpreting change scores for pain and functional status in low back pain: towards international consensus regarding minimal important change. *Spine*. Jan 1 2008;33(1):90-4. doi:10.1097/BRS.0b013e31815e3a10

4. Mannion AF, Balague F, Pellise F, Cedraschi C. Pain measurement in patients with low back pain. *Nature clinical practice Rheumatology*. Nov 2007;3(11):610-8. doi:10.1038/ncprheum0646

5. Jensen MP, Turner JA, Romano JM, Fisher LD. Comparative reliability and validity of chronic pain intensity measures. *Pain*. Nov 1999;83(2):157-62.

6. Deyo RA, Dworkin SF, Amtmann D, et al. Report of the NIH Task Force on Research Standards for Chronic Low Back Pain. *The spine journal : official journal of the North American Spine Society*. Jun 17 2014;doi:10.1016/j.spinee.2014.05.002

7. Hazard RG, Spratt KF, McDonough CM, et al. The impact of personal functional goal achievement on patient satisfaction with progress one year following completion of a functional restoration program for chronic disabling spinal disorders. *Spine*. 2009;34(25):2797.

8. Hazard RG, Spratt KF, McDonough CM, et al. Patient-centered evaluation of outcomes from rehabilitation for chronic disabling spinal disorders: the impact of personal goal achievement on patient satisfaction. *The Spine Journal*. 2012;12(12):1132-1137.

9. Sitzia J. How valid and reliable are patient satisfaction data? An analysis of 195 studies. *International Journal for Quality in Health Care*. 1999;11(4):319-328.

10. Kamper SJ, Ostelo RW, Knol DL, Maher CG, de Vet HC, Hancock MJ. Global Perceived Effect scales provided reliable assessments of health transition in people with musculoskeletal disorders, but ratings are strongly influenced by current status. *Journal of clinical epidemiology*. Jul 2010;63(7):760-766 e1. doi:10.1016/j.jclinepi.2009.09.009

11. Kamper SJ, Stanton TR, Williams CM, Maher CG, Hush JM. How is recovery from low back pain measured? A systematic review of the literature. *European spine journal : official publication of the European Spine Society, the European Spinal Deformity Society, and the European Section of the Cervical Spine Research Society*. Jan 2011;20(1):9-18. doi:10.1007/s00586-010-1477-8

12. Kamper SJ, Maher CG, Mackay G. Global rating of change scales: a review of strengths and weaknesses and considerations for design. *The Journal of manual & manipulative therapy*. 2009;17(3):163-70.

13. Turner-Stokes L. Goal attainment scaling (GAS) in rehabilitation: a practical guide. *Clinical rehabilitation*. 2009;

14. Kiresuk TJ, Sherman MRE. Goal attainment scaling: A general method for evaluating comprehensive community mental health programs. *Community mental health journal*. 1968;4(6):443-453.

15. Hurn J, Kneebone I, Cropley M. Goal setting as an outcome measure: a systematic review. *Clinical rehabilitation*. 2006;20(9):756-772.

16. Mannion AF, Caporaso F, Pulkovski N, Sprott H. Goal attainment scaling as a measure of treatment success after physiotherapy for chronic low back pain. *Rheumatology (Oxford, England)*. Sep 2010;49(9):1734-8. doi:10.1093/rheumatology/keq160
